# The Tumour Response to Induction Chemotherapy has Prognostic Value for Long-Term Survival Outcomes after Intensity-Modulated Radiation Therapy in Nasopharyngeal Carcinoma

**DOI:** 10.1038/srep24835

**Published:** 2016-04-21

**Authors:** Hao Peng, Lei Chen, Yuan Zhang, Wen-Fei Li, Yan-Ping Mao, Xu Liu, Fan Zhang, Rui Guo, Li-Zhi Liu, Li Tian, Ai-Hua Lin, Ying Sun, Jun Ma

**Affiliations:** 1 Department of Radiation Oncology, Sun Yat-sen University Cancer Center, State Key Laboratory of Oncology in Southern China, Collaborative Innovation Center for Cancer Medicine, People’s Republic of China; 2 Imaging Diagnosis and Interventional Center, Sun Yat-sen University Cancer Center, State Key Laboratory of Oncology in Southern China, Collaborative Innovation Center for Cancer Medicine, People’s Republic of China; 3 Department of Medical Statistics and Epidemiology, School of Public Health, Sun Yat-sen University, People’s Republic of China

## Abstract

The prognostic value of the tumour response to induction chemotherapy (IC) for long-term survival outcomes after intensity-modulated radiation therapy in nasopharyngeal carcinoma (NPC) remains unknown. We retrospectively reviewed 1811 consecutive patients with newly diagnosed NPC treated using IMRT, and 399 eligible patients with pre- and post-induction chemotherapy magnetic resonance images were recruited. The clinicopathological features of patients with different tumour responses were compared using the Chi-square test or Fisher’s exact test. Prognostic value was assessed using a multivariate Cox proportional hazards model. After IC, 101/399 (25.3%) patients had a complete tumour response overall (CR), 262 (65.7%) had a partial response (PR) and 36 (9.0%) had stable disease (SD). The 4-year disease-free survival (DFS), overall survival (OS), distant metastasis-free survival (DMFS) and locoregional relapse-free survival (LRRFS) rates for CR vs. PR vs. SD were 90.0% vs. 79.0% vs. 58.2% (CR vs. PR: *P1* = 0.007; CR vs. SD: *P2* < 0.001; PR vs. SD: *P3* = 0.004), 95.7% vs. 88.7% vs. 70.2% (*P1* = 0.017, *P2* < 0.001, *P3* = 0.005), 92.0% vs. 87.4% vs. 74.3% (*P1* = 0.162, *P2* = 0.005, *P3* = 0.029) and 95.9% vs. 88.8% vs. 81.8% (*P1* = 0.024, *P2* = 0.006, *P3* = 0.268), respectively. Multivariate analysis identified that the tumour response to IC was an independent prognostic factor for DFS, OS and LRRFS.

Nasopharyngeal carcinoma (NPC) is a cancer with an extremely unbalanced geographical distribution, with an age standardised incidence rate of 20–50 per 100,000 males in south China^1^. Differentiated tumours with surface keratin are defined as type I, whereas type II/III refer to non-keratinising carcinoma which is the main pathology type in south China. Due to the anatomic constraints and a high degree of radiosensitivity, radiotherapy (RT) is the primary and only curative treatment for NPC. Previous studies showed that patients with advanced disease could benefit from chemotherapy^2^,^3^,^4^,^5^,^6^, and concurrent chemoradiotherapy (CCRT) thereafter was established as the main standard treatment for advanced NPC by National Comprehensive Cancer Network (NCCN) guidelines.

The value of additional induction chemotherapy (IC) before CCRT in advanced NPC has received intense investigation. In 2009, Hui *et al.* reported that neoadjuvant docetaxel-cisplatin followed by CCRT provided a 3-year overall survival (OS) benefit in stage III-IVB NPC^7^. Regretfully, other subsequent induction chemotherapy regimens followed by CCRT have failed to demonstrate any OS benefits compared to RT with CCRT or RT alone in prospective clinical trials^8^,^9^,^10^,^11^,^12^.

However, despite these negative outcomes, IC may have potential clinical value. Previous studies reported that the response to chemotherapy correlate with clinical outcome^13^,^14^,^15^. Most recently, Liu *et al.* revealed that the unsatisfactory tumour response after induction chemotherapy could predict poor prognosis for patients with advanced-stage NPC^16^. However, the sample was relative small. Moreover, the prognostic difference was only discussed between stable/progressive disease (SD/PD) and complete/partial response (CR/PR) groups, and was not investigated between CR and PR groups. Therefore, on the basis of this premise, we conducted a retrospective study to further analyse the prognostic value of different tumour responses to induction chemotherapy in NPC patients who received intensity-modulated radiation therapy (IMRT).

## Materials and Methods

### Patient Selection

Of the 1811 patients with newly diagnosed non-metastatic NPC treated between November 2009 and February 2012 at Sun Yat-sen University Cancer Center, and the 399 patients for whom both pre- and post-induction chemotherapy magnetic resonance (MR) images of the nasopharynx and cervical region were available were retrospectively analysed. This study was conducted in compliance with the institutional policy regarding the protection of patients’ private information and approved by the Research Ethics Committee of Sun Yat-sen University Cancer Center. All the methods were carried out in accordance with the approved guidelines of Sun Yat-sen University Cancer Center. Written informed consent was obtained from all patients prior to therapy.

### Clinical Staging

Routine staging workup included a complete history, clinical examinations of the head and neck, direct fibre-optic nasopharyngoscopy, magnetic resonance imaging (MRI) of skull base and whole neck, chest radiography, whole-body bone scan, abdominal sonography, and positron emission tomography-CT if clinically-indicated. Immunoglobulin A antibodies against EBV viral caspid antigen (VCA-IgA) and Epstein Barr virus early antigen (EA-IgA) were quantified. All patients underwent dental evaluations before RT.

Patients were restaged according to the 7^th^ edition of the International Union against Cancer/American Joint Committee on Cancer (UICC/AJCC) staging system^17^. All MRI and clinical records were reviewed to minimize heterogeneity in restaging. Two radiologists evaluated all scans separately, and disagreements were resolved by consensus.

### Imaging Protocol

All patients underwent MRI of the region from the suprasellar cistern to the inferior margin at the sternal end of the clavicle using a head-and-neck coil with a 3 Tesla system (Trio Tim; Siemens, Erlangen, Germany). T1-weighted fast spin-echo images in the axial, coronal and sagittal planes (repetition time [TR]/echo time [TE] = 650 ms/9 ms), T2-weighted fast spin-echo MR images in the axial plane (TR/TE = 2470 ms/90 ms) and a spin-echo echo-planar DWI sequence (matrix = 192 × 192; TR/TE, 5100 ms/96 ms; *b*-values = 0 and 1000 s/mm^2^; three signal averages) were obtained before contrast injection. After intravenous administration of gadopentetate dimeglumine (0.1 mmol/kg body weight; Magnevist, Schering, Berlin, Germany), axial and sagittal T1-weighted spin-echo and coronal T1-weighted fat-suppressed spin-echo sequences were performed (same parameters as without contrast; 5 mm-thick sections with 1 mm interslice gap for axial plane, 6 mm-thick sections with 1 mm interslice gap for coronal and sagittal planes; matrix size, 512 × 512).

### Diagnostic Criteria for Tumour Response

Tumour response was assessed using MRI images after completion of IC based on Response Evaluation Criteria in Solid Tumours (RECIST)^18^. Complete response (CR) was defined as disappearance of target lesion (short radius of target neck pathological lymph nodes <10 mm, short radius of retropharyngeal lymph nodes <5 mm). Partial response (PR) was defined as a reduction of at least 30% in the sum of longest diameter of target lesions (using baseline sum longest diameter as reference). Progressive disease (PD) was defined as an increase of at least 20% in the sum of longest diameter of target lesions (using smallest sum longest diameter since treatment started or appearance of one or more new lesions as reference). Stable disease (SD) was defined as neither sufficient shrinkage to qualify for PR nor sufficient increase to qualify for PD (using smallest sum longest diameter since treatment started as reference). Overall tumor response, local tumor response and regional tumor response was defined as per Table 1.

## Treatment

### Radiotherapy

All patients underwent IMRT while immobilized using a custom head-to-neck thermoplastic cast with the patient’s neck resting on a support. A high-resolution contrast planning CT scan was taken from the vertex to 2 cm below sternoclavicular joint (slice thickness, 3 mm). Target volumes were delineated slice-by-slice on treatment planning CT scans using an individualized delineation protocol that complies with International Commission on Radiation Units and Measurements reports 50 and 62. Prescribed doses were 66–72 Gy at 2.12–2.43 Gy/fraction to planning target volume (PTV) of primary gross tumour volume (GTVnx), 64–70 Gy to PTV of GTV of involved lymph nodes (GTVnd), 60–63 Gy to PTV of high-risk clinical target volume (CTV1), and 54–56 Gy to PTV of low-risk clinical target volume (CTV2). All targets were treated simultaneously using the simultaneous integrated boost technique.

### Chemotherapy

According to institutional guidelines, we recommended RT alone for stage I, concurrent chemoradiotherapy (CCRT) for stage II, and CCRT +/− IC/adjuvant chemotherapy (ACT) for stage III to IVA-B. IC consisted of cisplatin (80 mg/m^2^) with 5-fluorouracil (1000 mg/m^2^) (PF), cisplatin (75 mg/m^2^) with docetaxel (75 mg/m^2^) (TP), or cisplatin (60 mg/m^2^) with 5-fluorouracil (600 mg/m^2^) and docetaxel (60 mg/m^2^) (TPF) every three weeks for two or three or more cycles. Concurrent chemotherapy was cisplatin weekly (30–40 mg/m^2^) or on weeks 1, 4 and 7 (80–100 mg/m^2^) of radiotherapy.

### Follow-Up and Statistical Analysis

Follow-up was measured from first day of therapy to last examination or death. Patients were followed by MRI and plasma EBV DNA at least every 3 months during first 2 years, then every 6 months thereafter (or until death). The end points (time to first defining event) were disease-free survival (DFS), overall survival (OS), distant metastasis-free survival (DMFS), locoregional relapse-free survival (LRRFS), local relapse-free survival (LRFS) and regional relapse-free survival (RRFS).

The Chi-square test or Fisher’s exact test were used to compare categorical variables and treatment failure patterns between the CR, PR and SD groups. Life-table estimation was performed using the Kaplan-Meier method and log-rank test. The multivariate Cox proportional hazards model was used to estimate hazard ratios (HRs) and 95% confidence intervals (CIs); age, gender, smoking, drinking, pathological type, T category, N category, concurrent chemotherapy, overall response were included as variables. All tests were two-sided; *P* < 0.05 was considered significant. Stata Statistical Package 12 (StataCorp LP, College Station, TX, USA) was used for all analyses.

## Results

### Patient Characteristics

For the 399 patients, the male (*n* = 308)-to-female (*n* = 91) ratio was 3.4:1. The median age for the whole cohort was 45 years (range, 14–76 years). The baseline characteristics of patients were list in Table 2. Sixty-eight (17.0%) patients did not received concurrent chemotherapy with 16 (23.5%) in CR group, 45 (66.2%) in PR group and 7 (10.3%) in SD group, and no significant difference was found between the three groups with respect to the omission of concurrent chemotherapy.

The overall tumour response to IC was a CR for 101/399 (25.3%) patients, PR for 262 (65.7%) patients, and SD for 36 (9.0%) patients. No patients had PD after IC. The local tumour response was a CR for 139/399 (34.8%) patients, PR for 195 (48.9%) patients and SD for 65 (16.3%) patients. Nineteen (4.8%) patients had N0 disease and were not included in the analysis of regional response. With regards to regional tumour response, 167/380 (43.9%), 166/380 (43.7%) and 47380 (12.4%) patients had a CR, PR and SD, respectively.

Significantly more patients with SD were previous smokers (58.3% vs. 29.7%, *P* = 0.002; 58.3% vs. 40.1%, *P* = 0.038) and had advanced T category (94.4% vs. 73.3%, *P* = 0.008; 94.4% vs. 80.5%, *P* = 0.041) compared to patients who achieved a CR or PR. Obviously, patients with CR received more cycles of induction chemotherapy compared with that of patients with PR (*P* = 0.009) or SD (*P* < 0.001). No significant associations were observed between any other clinicopathological feature and the overall tumour response.

### Treatment Failure Patterns

Median follow-up time for the entire cohort was 49.9 months (range, 1.3–76.4 months). By final follow-up, 21/399 (5.3%) patients had developed local failure, 23/399 (5.8%) patients developed regional failure and 51/399 (12.8%) patients developed distant failure.

With regards to overall tumour response after induction chemotherapy, patients with SD had higher rates of local failure (11.1% vs. 2.0%, *P* = 0.022) and distant failure (25% vs. 7.9%, *P* = 0.008) after IMRT than patients with a CR (Table 3). The distant failure rates of patients with overall PR and SD after induction chemotherapy were almost significantly different (13.0% vs. 25%, *P* = 0.054).

Patients with local SD after induction chemotherapy had higher rates of local failure after IMRT compared to patients with a local CR (10.8% vs. 2.2%, *P* = 0.008). The local failure rates of the local PR and CR groups were almost significantly different (6.7% vs. 2.2%, *P* = 0.057). Patients with a regional CR after induction chemotherapy had a lower rate of distant failure after IMRT than patients with a regional PR (7.2% vs. 14.5%, *P* = 0.033) and regional SD (7.2% vs. 25.5%, *P* < 0.001). Additionally, patients with a regional CR had a lower rate of regional failure after IMRT than patients with a regional PR (2.4% vs. 8.4%, *P* = 0.015).

### Univariate Analysis

**Figure 1:**
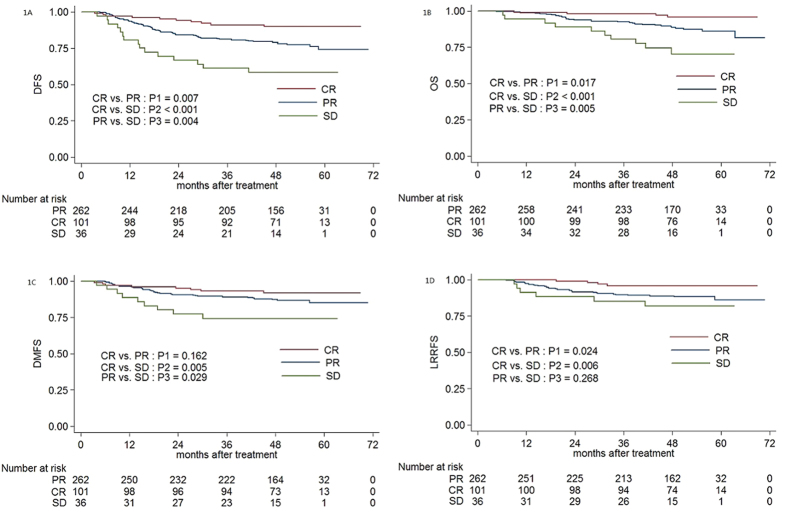
Kaplan-Meier DFS (**1A**), OS (**1B**), DMFS (**1C**) and LRRFS (**1D**) curves for 399 patients with NPC receiving IMRT stratified as the CR, PR and SD groups based on overall tumour response after induction chemotherapy. Abbreviations: DFS = disease-free survival; OS = overall survival; LRRFS = local-regional relapse-free survival; DMFS = distant metastasis-free survival. CR = complete response; PR = partial response; SD = stable disease.

The 4-year DFS, OS, DMFS and LRRFS rates for the entire cohort were 79.9%, 88.9%, 87.4% and 90.1%, respectively. In terms of the overall response after induction chemotherapy, the 4-year DFS, OS, DMFS and LRRFS rates for patients with CR vs. PR vs. SD were 90.0% vs. 79.0% vs. 58.2% (CR vs. PR: *P1* = 0.007; CR vs. SD: *P2* < 0.001; PR vs. SD: *P3* = 0.004), 95.7% vs. 88.7% vs. 70.2% (*P1* = 0.017, *P2* < 0.001, *P3* = 0.005), 92.0% vs. 87.4% vs. 74.3% (*P1* = 0.162, *P2* = 0.005, *P3* = 0.029) and 95.9% vs. 88.8% vs. 81.8% (*P1* = 0.024, *P2* = 0.006, *P3* = 0.268; Fig. 1), respectively.

**Figure 2:**
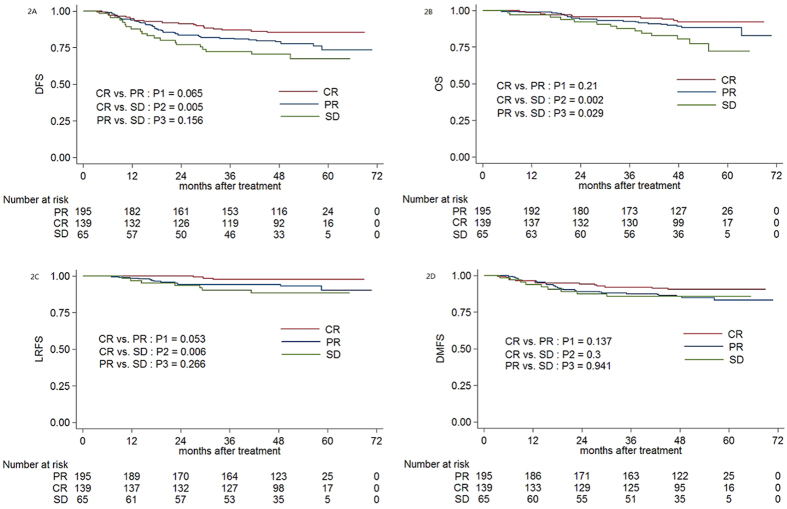
Kaplan-Meier DFS (**2A**), OS (**2B**), LRFS (**2C**) and DMFS (**2D**) curves for 399 patients with NPC receiving IMRT stratified as the CR, PR and SD groups based on the local tumour response after induction chemotherapy. Abbreviations: DFS = disease-free survival; OS = overall survival; LRFS = local relapse-free survival; DMFS = distant metastasis-free survival. CR = complete response; PR = partial response; SD = stable disease.

The 4-year DFS, OS, LRFS and DMFS rates for patients with a local CR vs. PR vs. SD after induction chemotherapy were 85.5% vs. 79.1% vs. 70.7% (CR vs. PR: *P1* = 0.065; CR vs. SD: *P2* = 0.005; PR vs. SD: *P3* = 0.156), 92.3% vs. 89.2% vs. 80.6% (*P1* = 0.21, *P2* = 0.002, *P3* = 0.029), 97.7% vs. 94.2% vs. 88.5% (*P1* = 0.053, *P2* = 0.006, *P3* = 0.266) and 90.5% vs. 85.7% vs. 85.9% (*P1* = 0.137, *P2* = 0.3, *P3* = 0.941; Fig. 2), respectively.

**Figure 3:**
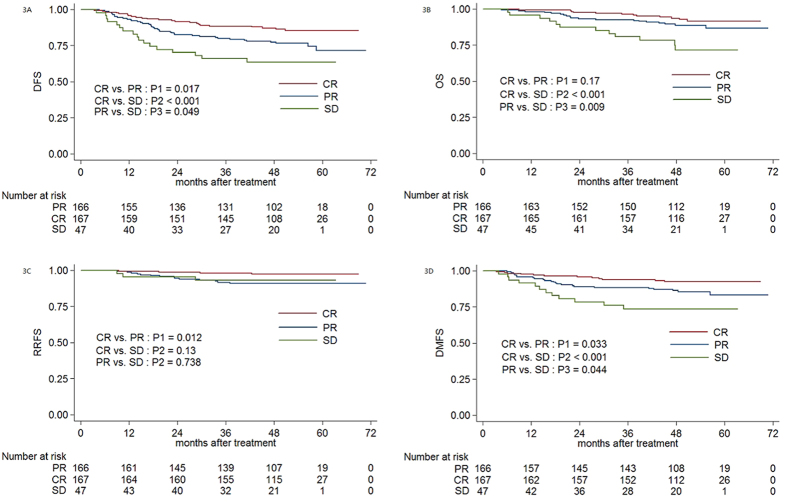
Kaplan-Meier DFS (**3A**), OS (**3B**), RRFS (**3C**) and DMFS (**3D**) curves for 399 patients with NPC receiving IMRT stratified as the CR, PR and SD groups based on the regional tumour response after induction chemotherapy. Abbreviations: DFS = disease-free survival; OS = overall survival; RRFS = regional relapse-free survival; DMFS = distant metastasis-free survival. CR = complete response; PR = partial response; SD = stable disease.

The 4-year DFS, OS, RRFS and DMFS rates for patients with a regional CR vs. PR vs. SD after induction chemotherapy were 87.2% vs. 77.4% vs. 63.5% (CR vs. PR: *P1* = 0.017; CR vs. SD: *P2* < 0.001; PR vs. SD:*P3* = 0.049), 93.7% vs. 88.7% vs. 71.5% (*P1* = 0.17, *P2* < 0.001, *P3* = 0.009), 97.5% vs. 91.1% vs. 93.1% (*P1* = 0.012, *P2* = 0.13, *P3* = 0.738) and 92.6% vs. 86.4% vs. 73.5% (*P1* = 0.033, *P2* < 0.001, *P3* = 0.044; Fig. 3), respectively.

### Multivariate Analysis

Multivariate analysis was performed to adjust for potential prognostic factors, including age, gender, smoking, drinking, pathological type, T category, N category and concurrent chemotherapy. Consistent with the results of univariate analysis, the overall tumour response after induction chemotherapy was an independent prognostic factor for DFS, OS and LRRFS (Table 4).

## Discussion

The outcomes of this current study revealed that satisfactory tumour response to IC was associated with significantly improved DFS, OS and LRRFS for patients with NPC in the era of IMRT. The reported rates of overall CR, PR, and SD after induction chemotherapy range from 8% to 27%, 55% to 64%, and 11% to 17.6%, respectively^7^,^8^,^19^,^20^, and the overall tumour responses of the patients in this study are consistent with these results. Liu *et al.*^16^ proved that patients with SD had poorer prognosis compared with patients with CR/PR. However, the prognostic difference was not clear between CR and PR groups. In this larger cohort study, we provided more detailed results about the prognostic value of different tumour response to induction chemotherapy.

Of note, the SD group had a higher percentage of smoking compared with that of CR or PR group, and this may influenced the survival outcomes since smoking was previously reported as a prognostic factor in advanced NPC^21^. Moreover, more patients in the SD group had T3-4 stage disease compared with that of patients in CR or PR group. This unbalanced distribution of host and tumour factors between the three groups may influence the significant results of univariate analysis for DMFS since multivariate analysis did not identify tumour response as an independent prognostic factor for DMFS. On multivariate analysis of LRRFS, patients with CR had better LRFFS compared with patients with PR. However, this significant difference did not exist between CR/PR and SD groups. Reasonable explanation was that only 6 (16.7%) patients in the SD group experienced locoregional failure. Therefore, the small sample resulted in a low power to detect significant differences between the CR/PR and SD groups. In addition, 68 (17.0%) patients did not received concurrent chemotherapy after induction chemotherapy. The main reason for the omission of concurrent chemotherapy was that patients had poor status and could not tolerate concurrent chemotherapy.

NPC is highly chemosensitive and radiosensitive. Although most patients with advanced NPC respond well to chemotherapy, recurrence of distant metastases is the major cause of treatment failure and has a poor prognosis^22^. Patients with SD may develop resistance to anticancer drugs which is a major factor that leads to this pattern of failure^23^. A number of molecular mechanisms have been linked to chemoresistance and poor prognosis. For example, positive tumour expression of multidrug resistance 1 (MDR1) is associated with poor overall survival in patients with recurrent or metastatic NPC receiving chemotherapy^23^. Multidrug resistance-associated protein-1 (MRP1) expression was identified as an independent prognostic factor for 5-year overall survival and disease-free survival^24^. Moreover, upregulation of the drug-resistance gene annexin I (*ANX-I*) is associated with a poorer prognosis in NPC, breast cancer and gastric cancer^25^, and radio- and chemo-sensitivity could be induced by suppressing expression of Jab1/CSN5^26^. These molecular mechanisms may have potential as therapeutic targets to improve the prognosis of patients with distant metastasis.

Despite the outcome that unsatisfactory (PR or SD) tumour response after IC indicated poor prognosis, we should pay attention to the factors which may affect the result. Firstly, using the RECIST standard^18^, it is difficult to define the local response in advanced-stage NPC with skull base invasion. The imaging changes in the skull base are not as obvious as changes in the nasopharynx tumour or neck lymph nodes, which may lead to inaccurate evaluation of local tumour response. More appropriate evaluation criteria for defining the tumour responses in NPC should be established. Secondly, the patients in this retrospective study received different induction chemotherapy regimens and varying numbers of cycles: 60.4% (61/101) of patients in CR group received equal or more than 3 cycles of induction chemotherapy, comparing with 45.0% (118/262) in PR group and 25.0% (9/36) in SD group. This should be reasonable because clinicians would quit IC and turn to RT immediately if the evaluation after two cycles IC was SD. Therefore, patients in SD group would receive less cycles of IC. The heterogeneity of IC regimens should be small and would not influence the results because most patients in the study received the standard treatment regimens according the NCCN guidelines.

The current study showed that patients with unsatisfactory tumour responses after IC have poorer prognosis compared with that of patients with satisfactory tumour responses. Therefore, we should pay more attention to patients with SD after induction chemotherapy in clinical practice. More intensive treatment regimens like higher radiation dose and adjuvant chemotherapy should be considered. Moreover, regular follow-up should also be provided to detect early recurrence and reduce the potential risk of distant failure.

This study is limited by its retrospective nature and fact the follow-up time may have been insufficient, though we selected DFS as the major endpoint to address this shortcoming. Moreover, many other prognostic factors including pre-treatment plasma Epstein-Barr virus DNA load^27^,^28^,^29^, primary tumour volume^30^,^31^ and the pre-treatment serum lactate dehydrogenase (LDH) level^32^ were not assessed in this study. Of note, only 2 (0.5%) patients had type I NPC, which is characteristic of endemic NPC areas and hence these results might not apply to other non-endemic regions. Further prospective studies of larger cohorts are warranted to confirm the prognostic value of the tumour response to induction chemotherapy in patients with NPC treated with IMRT.

## Conclusions

Tumour response to induction chemotherapy is an independent prognostic factor for patients with NPC receiving IMRT. Assessing tumour response after induction chemotherapy may assist prognostication and refine the treatment of high-risk patients with NPC. The outcomes in this current study need to be confirmed by prospective studies with large cohorts.

## Additional Information

**How to cite this article**: Peng, H. *et al.* The Tumour Response to Induction Chemotherapy has Prognostic Value for Long-Term Survival Outcomes after Intensity-Modulated Radiation Therapy in Nasopharyngeal Carcinoma. *Sci. Rep.* **6**, 24835; doi: 10.1038/srep24835 (2016).

## Figures and Tables

**Table 1:** Overall tumour responses for various local and regional tumour responses with or without the appearance of new lesions.

**Local lesions**	**Regional lesions**	**New lesions**	**Overall response**
CR	CR	No	CR
CR	PR/SD	No	PR
PR	Non-PD	No	PR
SD	CR/PR	No	PR
SD	SD	No	SD
PD	Any	Yes or No	PD
Any	PD	Yes or No	PD
Any	Any	Yes	PD

Abbreviations: CR = complete response; PR = partial response; SD = stable disease; PD = progressive disease.

**Table 2:** Association of clinicopathological features with the overall tumour response after induction chemotherapy for the 399 patients with advanced stage NPC.

Characteristic	CR	PR	SD	*P1*^b^	*P2*^b^	*P3*^b^
	No. (%)	No. (%)	No. (%)			
Age (years)				0.135	0.721	0.543
<50	76 (75.2)	176 (67.2)	26 (72.2)			
≥50	25 (24.8)	86 (32.8)	10 (27.8)			
Sex				0.955	0.106	0.081
Male	77 (76.2)	199 (76.0)	32 (88.9)			
Female	24 (23.8)	63 (24.0)	4 (11.1)			
Smoking				0.067	0.002	0.038
Yes	30 (29.7)	105 (40.1)	21 (58.3)			
No	71 (70.3)	157 (59.9)	15 (41.7)			
Drinking				0.802	0.091	0.083
Yes	11 (10.9)	31 (11.8)	8 (22.2)			
No	90 (89.1)	231 (88.2)	28 (77.8)			
WHO pathology						
Type I	0 (0)	2 (0.8)	0 (0)	1.000	-	1.000
Type II/III	101 (100)	260 (99.2)	36 (100)			
T category^a^				0.131	0.008	0.041
T1-2	27 (26.7)	51 (19.5)	2 (5.6)			
T3-4	74 (73.3)	211 (80.5)	34 (94.4)			
N category^a^				0.471	0.728	0.387
N0-1	65 (64.4)	179 (68.3)	22 (61.1)			
N2-3	36 (35.6)	83 (31.7)	14 (38.9)			
Overall stage^a^				0.397	0.11	0.184
II	16 (15.8)	28 (10.7)	1 (2.8)			
III	42 (41.6)	113 (43.1)	19 (52.8)			
IVA-IVB	43 (42.6)	121 (46.2)	16 (44.4)			
Concurrent chemotherapy				0.761	0.619	0.737
Yes	85 (84.2)	217 (82.8)	29 (80.6)			
No	16 (15.8)	45 (17.2)	7 (19.4)			
Cycles of IC				0.009	<0.001	0.023
2	40 (39.6)	144 (55.0)	27 (75.0)			
≥3	61 (60.4)	118 (45.0)	9 (25.0)			

Abbreviations: CR = complete response; PR = partial response; SD = stable disease; WHO = World Health Organization; NPC = nasopharyngeal carcinoma; IC = induction chemotherapy.

^a^According to the 7^th^ edition of the AJCC/UICC staging system.

^b^*P-*values were calculated using the Chi-square test or Fisher’s exact test.

*P1*-CR vs. PR; *P2*-CR vs. SD; *P3*-PR vs. SD.

**Table 3:** Patterns of treatment failure for the 399 patients with NPC stratified by tumour response after induction chemotherapy.

Tumour response	Local failure	Regional failure	Distant failure
	No. (%)	No. (%)	No. (%)
Overall response			
CR	2 (2.0)	2 (2.0)	8 (7.9)
PR	17 (6.5)	17 (6.5)	34 (13.0)
SD	4 (11.1)	2 (5.6)	9 (25)
*P1*	0.084	0.084	0.177
*P2*	0.022	0.274	0.008
*P3*	0.34	0.827	0.054
Local response			
CR	3 (2.2)	6 (4.3)	13 (9.4)
PR	13 (6.7)	12 (6.2)	29 (14.9)
SD	7 (10.8)	3 (4.6)	9 (13.8)
*P1*	0.057	0.464	0.134
*P2*	0.008	0.923	0.335
*P3*	0.282	0.637	0.839
Regional response			
CR	10 (6.0)	4 (2.4)	12 (7.2)
PR	8 (4.8)	14 (8.4)	24 (14.5)
SD	3 (6.4)	3 (6.4)	12 (25.5)
*P1*	0.637	0.015	0.033
*P2*	0.92	0.175	<0.001
*P3*	0.676	0.647	0.074

Abbreviations: NPC = nasopharyngeal carcinoma; CR = complete response; PR = partial response; SD = stable disease.

*P1*-CR vs. PR; *P2*-CR vs. SD; *P3*-PR vs. SD.

**Table 4:** Multivariate analysis of prognostic factors associated with clinical outcomes based on the overall tumour responses after induction chemotherapy.

Endpoint	Variable	*P*^a^	HR	95% CI for HR
DFS	Overall response, CR vs. PR	0.008	0.403	0.206–0.788
	Overall response, SD vs. PR	0.005	2.288	1.289–4.061
OS	Overall response, CR vs. PR	0.024	0.301	0.106–0.853
	Overall response, SD vs. PR	0.023	2.294	1.119–4.702
	N category	0.003	2.431	1.357–4.353
DMFS	N category	<0.001	2.808	1.614–4.886
LRRFS	Overall response, CR vs. PR	0.032	0.319	0.112–0.904
	Gender	0.015	2.240	1.173–4.278

Abbreviations: DFS = disease-free survival; OS = overall survival; DMFS = distant metastases-free survival; LRRFS = locoregional relapse-free survival; HR = hazard ratio; CI = confidence interval; CR = complete response; PR = partial response; SD = stable disease.

^a^Multivariate *P*-values were calculated using an adjusted Cox proportional-hazards model. The following parameters were included in the Cox proportional hazards model with backward elimination: age (≥50 y vs. <50 y), gender (male vs. female), smoking (yes vs. no), drinking (yes vs. no), pathological type (type I vs. type II/III), T category, N category, concurrent chemotherapy (yes vs. no), overall tumour response (CR vs. PR; SD vs. PR).

## References

[b1] Jemal A., Bray F. & Center M. M. *Global cancer statistics.* CA Cancer J Clin 61(2), p. 69–90 (2011). 10.3322/caac.20107 21296855

[b2] Al-Sarraf M., LeBlanc M. & Giri P. G. Chemoradiotherapy versus radiotherapy in patients with advanced nasopharyngeal cancer: phase III randomized Intergroup study 0099. J Clin Oncol. 16(4), p. 1310–7 (1998). 9552031 10.1200/JCO.1998.16.4.1310

[b3] Chan A. T., Leung S. F. & Ngan R. K. Overall survival after concurrent cisplatin-radiotherapy compared with radiotherapy alone in locoregionally advanced nasopharyngeal carcinoma. J Natl Cancer Inst. 97(7), p. 536–9 (2005). 15812080 10.1093/jnci/dji084

[b4] Lee A. W., Lau W. H. & Tung S. Y. Preliminary results of a randomized study on therapeutic gain by concurrent chemotherapy for regionally-advanced nasopharyngeal carcinoma: NPC-9901 Trial by the Hong Kong Nasopharyngeal Cancer Study Group. J Clin Oncol. 23(28), p. 6966–75 (2005). 16192584 10.1200/JCO.2004.00.7542

[b5] Lin J. C., Jan J. S. & Hsu C. Y. Phase III study of concurrent chemoradiotherapy versus radiotherapy alone for advanced nasopharyngeal carcinoma: positive effect on overall and progression-free survival. J Clin Oncol. 21(4), p. 631–7 (2003). 12586799 10.1200/JCO.2003.06.158

[b6] Wee J., Tan E. H. & Tai B. C. Randomized trial of radiotherapy versus concurrent chemoradiotherapy followed by adjuvant chemotherapy in patients with American Joint Committee on Cancer/International Union against cancer stage III and IV nasopharyngeal cancer of the endemic variety. J Clin Oncol. 23(27), p. 6730–8 (2005). 16170180 10.1200/JCO.2005.16.790

[b7] Hui E. P., Ma B. B. & Leung S. F. Randomized phase II trial of concurrent cisplatin-radiotherapy with or without neoadjuvant docetaxel and cisplatin in advanced nasopharyngeal carcinoma. J Clin Oncol. 27(2), p. 242–9 (2009). 19064973 10.1200/JCO.2008.18.1545

[b8] Fountzilas G., Ciuleanu E. & Bobos M. Induction chemotherapy followed by concomitant radiotherapy and weekly cisplatin versus the same concomitant chemoradiotherapy in patients with nasopharyngeal carcinoma: a randomized phase II study conducted by the Hellenic Cooperative Oncology Group (HeCOG) with biomarker evaluation. Ann Oncol. 23(2), p. 427–35 (2012). 21525406 10.1093/annonc/mdr116

[b9] Lee A. W., Ngan R. K. & Tung S. Y. Preliminary results of trial NPC-0501 evaluating the therapeutic gain by changing from concurrent-adjuvant to induction-concurrent chemoradiotherapy, changing from fluorouracil to capecitabine, and changing from conventional to accelerated radiotherapy fractionation in patients with locoregionally advanced nasopharyngeal carcinoma. Cancer 121(8), p. 1328–38 (2015). 25529384 10.1002/cncr.29208

[b10] Hareyama M., Sakata K. & Shirato H. A prospective, randomized trial comparing neoadjuvant chemotherapy with radiotherapy alone in patients with advanced nasopharyngeal carcinoma. Cancer 94(8), p. 2217–23 (2002). 12001120 10.1002/cncr.10473

[b11] International Nasopharynx Cancer Study, G. and V.I. Trial. Preliminary results of a randomized trial comparing neoadjuvant chemotherapy (cisplatin, epirubicin, bleomycin) plus radiotherapy vs. radiotherapy alone in stage IV(> or = N2, M0) undifferentiated nasopharyngeal carcinoma: a positive effect on progression-free survival. Int J Radiat Oncol Biol Phys. 35(3), p. 463–9 (1996). 8655368 10.1016/s0360-3016(96)80007-1

[b12] Ma J., Mai H. Q. & Hong M. H. Results of a prospective randomized trial comparing neoadjuvant chemotherapy plus radiotherapy with radiotherapy alone in patients with locoregionally advanced nasopharyngeal carcinoma. J Clin Oncol. 19(5), p. 1350–7 (2001). 11230478 10.1200/JCO.2001.19.5.1350

[b13] Bacci G., Longhi A. & Versari M. Prognostic factors for osteosarcoma of the extremity treated with neoadjuvant chemotherapy: 15-year experience in 789 patients treated at a single institution. Cancer 106(5), p. 1154–61 (2006). 16421923 10.1002/cncr.21724

[b14] Picci P., Rougraff B. T. & Bacci G. Prognostic significance of histopathologic response to chemotherapy in nonmetastatic Ewing’s sarcoma of the extremities. J Clin Oncol. 11(9), p. 1763–9 (1993). 8355043 10.1200/JCO.1993.11.9.1763

[b15] Visser J. H., Wessels G. & Hesseling P. B. Prognostic value of day 14 blast percentage and the absolute blast index in bone marrow of children with acute lymphoblastic leukemia. Pediatr Hematol Oncol. 18(3), p. 187–91 (2001). 11293286 10.1080/08880010151114804

[b16] Liu L. T., Tang L. Q. & Chen Q. Y. The Prognostic Value of Plasma Epstein-Barr Viral DNA and Tumor Response to Neoadjuvant Chemotherapy in Advanced-Stage Nasopharyngeal Carcinoma. Int J Radiat Oncol Biol Phys. 93(4), p. 862–9 (2015). 26530755 10.1016/j.ijrobp.2015.08.003

[b17] Edge S. B. & Compton C. C. The American Joint Committee on Cancer: the 7th edition of the AJCC cancer staging manual and the future of TNM. Ann Surg Oncol. 17(6), p. 1471–4 (2010). 20180029 10.1245/s10434-010-0985-4

[b18] Therasse P., Arbuck S. G. & Eisenhauer E. A. New guidelines to evaluate the response to treatment in solid tumors. European Organization for Research and Treatment of Cancer, National Cancer Institute of the United States, National Cancer Institute of Canada. J Natl Cancer Inst. 92(3), p. 205–16 (2000). 10655437 10.1093/jnci/92.3.205

[b19] Airoldi M., Gabriele A. M. & Garzaro M. Induction chemotherapy with cisplatin and epirubicin followed by radiotherapy and concurrent cisplatin in locally advanced nasopharyngeal carcinoma observed in a non-endemic population. Radiother Oncol. 92(1), p. 105–10 (2009). 19264368 10.1016/j.radonc.2009.02.005

[b20] Golden D. W., Rudra S. & Witt M. E. Outcomes of induction chemotherapy followed by concurrent chemoradiation for nasopharyngeal carcinoma. Oral Oncol. 49(3), p. 277–82 (2013). 23102863 10.1016/j.oraloncology.2012.10.003

[b21] Guo S. S., Huang P. Y. & Chen Q. Y. The impact of smoking on the clinical outcome of locoregionally advanced nasopharyngeal carcinoma after chemoradiotherapy. Radiat Oncol. 9, p. 246 (2014). 25424191 10.1186/s13014-014-0246-y PMC4251838

[b22] Sun X., Su S. & Chen C. Long-term outcomes of intensity-modulated radiotherapy for 868 patients with nasopharyngeal carcinoma: an analysis of survival and treatment toxicities. Radiother Oncol. 110(3), p. 398–403 (2014). 24231245 10.1016/j.radonc.2013.10.020

[b23] Hsu C. H., Chen C. L. & Hong R. L. Prognostic value of multidrug resistance 1, glutathione-S-transferase-pi and p53 in advanced nasopharyngeal carcinoma treated with systemic chemotherapy. Oncology. 62(4), p. 305–12 (2002). 12138237 10.1159/000065061

[b24] Larbcharoensub N., Leopairat J. & Sirachainan E. Association between multidrug resistance-associated protein 1 and poor prognosis in patients with nasopharyngeal carcinoma treated with radiotherapy and concurrent chemotherapy. Hum Pathol. 39(6), p. 837–45 (2008). 18400250 10.1016/j.humpath.2007.10.009

[b25] Chow B. H., Chua D. T. & Sham J. S. Increased expression of annexin I is associated with drug-resistance in nasopharyngeal carcinoma and other solid tumors. Proteomics Clin Appl. 3(6), p. 654–62 (2009). 21136977 10.1002/prca.200800164

[b26] Pan Y., Zhang Q. & Atsaves V. Suppression of Jab1/CSN5 induces radio- and chemo-sensitivity in nasopharyngeal carcinoma through changes to the DNA damage and repair pathways. Oncogene. 32(22), p. 2756–66 (2013). 22797071 10.1038/onc.2012.294 PMC3566273

[b27] Chan A. T., Lo Y. M. & Zee B. Plasma Epstein-Barr virus DNA and residual disease after radiotherapy for undifferentiated nasopharyngeal carcinoma. J Natl Cancer Inst. 94(21), p. 1614–9 (2002). 12419787 10.1093/jnci/94.21.1614

[b28] Lin J. C., Wang W. Y. & Chen K. Y. Quantification of plasma Epstein-Barr virus DNA in patients with advanced nasopharyngeal carcinoma. N Engl J Med. 350(24), p. 2461–70 (2004). 15190138 10.1056/NEJMoa032260

[b29] Lo Y. M., Chan L. Y. & Chan A. T. Quantitative and temporal correlation between circulating cell-free Epstein-Barr virus DNA and tumor recurrence in nasopharyngeal carcinoma. Cancer Res. 59(21), p. 5452–5 (1999). 10554016

[b30] Guo R., Sun Y. & Yu X. L. Is primary tumor volume still a prognostic factor in intensity modulated radiation therapy for nasopharyngeal carcinoma? Radiother Oncol. 104(3), p. 294–9 (2012). 22998947 10.1016/j.radonc.2012.09.001

[b31] Tian Y. M., Xiao W. W. & Bai L. Impact of primary tumor volume and location on the prognosis of patients with locally recurrent nasopharyngeal carcinoma. Chin J Cancer 34(6), p. 247–53 (2015). 26063113 10.1186/s40880-015-0019-5 PMC4593352

[b32] Wan X. B., Wei L. & Li H. High pretreatment serum lactate dehydrogenase level correlates with disease relapse and predicts an inferior outcome in locally advanced nasopharyngeal carcinoma. Eur J Cancer 49(10), p. 2356–64 (2013). 23541571 10.1016/j.ejca.2013.03.008

